# 

**Published:** 1887-11

**Authors:** 


*Entered at the Post Office, at Austin, Texas, as Second-Class Mail Matter.*

Vol. III. NOVEMBER, 1887. No. 5.

LIBRARY

### DANIEL'S MEDICAL JOURNAL

Subscription, $2.00 a Year, in Advance.—Single Copies, Twenty-five Cents.

F. E. DANIEL, M. D., Editor and Publisher, AUSTIN, TEXAS.

**Northern Office, 1217 Filbert Street, Philadelphia.**

